# (*Z*)-Methyl 3-(4-ethoxy­anilino)but-2-enoate

**DOI:** 10.1107/S160053680800891X

**Published:** 2008-05-10

**Authors:** Li-Ping Zhang, Cai-Hua Ni, Zhi-Yong Li, Wei Zhang

**Affiliations:** a School of Chemical and Materials Engineering, Jiangnan University, 1800 Lihu Road, Wuxi 214122, Jiangsu, People’s Republic of China

## Abstract

The title compound, C_13_H_17_NO_3_, was synthesized from methyl 3-oxobutanoate and 4-ethoxy­benzenamine using a catalytic amount of InBr_3_ under solvent-free conditions. The 3-amino­but-2-enoic acid methyl ester group is planar and forms a dihedral angle of 83.4 (1)° with the benzene ring. The eth­oxy group is slightly twisted away from the benzene ring [dihedral angle = 13.8 (1)°]. An intra­molecular N—H⋯O hydrogen bond generating an *S*(6) ring is observed. Mol­ecules are linked into a chain along the *b* axis by inter­molecular C—H⋯O hydrogen bonding.

## Related literature

For general background on β-enamino esters, see: Bartoli *et al.* (1994 ▶); Cimarelli & Palmieri (1996 ▶); Cimarelli *et al.* (1994 ▶); Elassar & El-Khair (2003 ▶); Greenhill (1977 ▶); Lubell *et al.* (1991 ▶); Michael *et al.* (1999 ▶); Paola *et al.* (2000 ▶); Rybarczyk-Pirek & Grabowski (2002 ▶); Yunus *et al.* (2008 ▶).
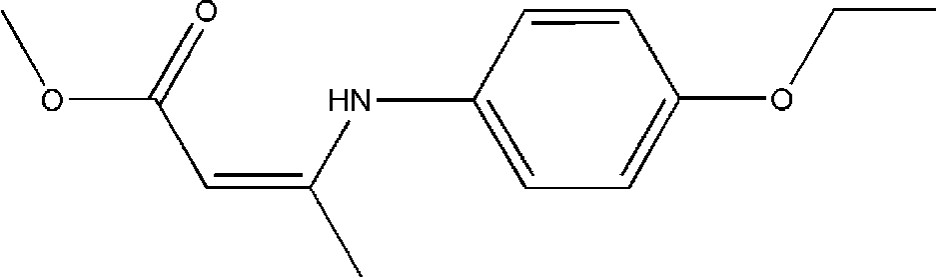


## Experimental

### 

#### Crystal data

C_13_H_17_NO_3_
*M* *_r_* = 235.28
Monoclinic, 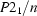

*a* = 12.421 (2) Å
*b* = 6.3372 (13) Å
*c* = 16.569 (3) Å
β = 96.519 (3)°
*V* = 1295.7 (4) Å^3^
*Z* = 4
Mo *K*α radiation
μ = 0.09 mm^−1^
*T* = 294 (2) K
0.30 × 0.26 × 0.20 mm

#### Data collection

Bruker SMART CCD area-detector diffractometer
Absorption correction: multi-scan (*SADABS*; Sheldrick, 1996 ▶) *T*_min_ = 0.942, *T*_max_ = 0.990
6917 measured reflections
2628 independent reflections
1629 reflections with *I* > 2σ(*I*)
*R*_int_ = 0.031

#### Refinement

*R*[*F* ^2^ > 2σ(*F* ^2^)] = 0.042
*wR*(*F* ^2^) = 0.136
*S* = 1.00
2628 reflections
158 parameters
H-atom parameters constrained
Δρ_max_ = 0.13 e Å^−3^
Δρ_min_ = −0.11 e Å^−3^

### 

Data collection: *SMART* (Bruker, 1998 ▶); cell refinement: *SAINT* (Bruker, 1999 ▶); data reduction: *SAINT*; program(s) used to solve structure: *SHELXS97* (Sheldrick, 2008 ▶); program(s) used to refine structure: *SHELXL97* (Sheldrick, 2008 ▶); molecular graphics: *SHELXTL* (Sheldrick, 2008 ▶); software used to prepare material for publication: *SHELXTL*.

## Supplementary Material

Crystal structure: contains datablocks global, I. DOI: 10.1107/S160053680800891X/ci2569sup1.cif

Structure factors: contains datablocks I. DOI: 10.1107/S160053680800891X/ci2569Isup2.hkl

Additional supplementary materials:  crystallographic information; 3D view; checkCIF report

## Figures and Tables

**Table 1:** Hydrogen-bond geometry (Å, °)

*D*—H⋯*A*	*D*—H	H⋯*A*	*D*⋯*A*	*D*—H⋯*A*
N1—H1⋯O2	0.86	2.08	2.741 (2)	133
C6—H6⋯O2^i^	0.93	2.57	3.362 (3)	143

Symmetry code: (i) 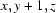.

**Fig. 1.**
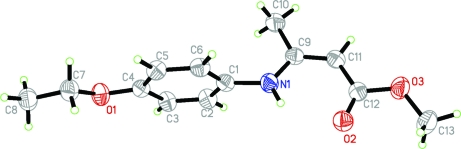
The molecular structure of the title compound, showing 30% probability displacement ellipsoids.

**Fig. 2.**
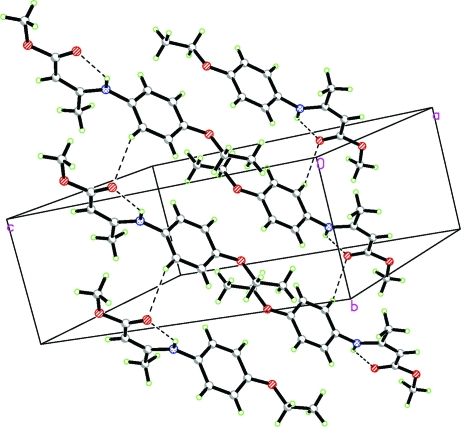
A view of the molecular packing in the title compound. Hydrogen bonds are shown as dashed lines.

## supplementary crystallographic information

### Comment

β-Enamino esters are useful precursors for the preparation of biologically active compounds such as β-enamino acids, γ-enamino alcohols or β-enamino esters (Lubell *et al.*, 1991; Bartoli *et al.*, 1994; Cimarelli *et al.*, 1994; Cimarelli & Palmieri, 1996). Therefore, many synthetic methods have been developed for the preparation of these compounds (Greenhill, 1977; Elassar *et al.*, 2003; Michael *et al.*, 1999). As part of our program on developing new environmental friendly methodologies for the preparation of β-enamino compounds, we have synthesized the title compound (Fig.1). We report here the crystal structure of it.

In the title molecule, the 3-amino-but-2-enoic acid methyl ester group is planar (r.m.s. deviation 0.045 Å) and it forms a dihedral angle of 83.4 (1)° with the benzene ring. The ethoxy group is slightly twisted away from the benzene ring [dihedral angle 13.8 (1)°]. An intramolecular N1—H1···O2 hydrogen bond generating an S(6) ring is observed. The N1—C9 bond length [1.341 (2) Å] is shorter than the N1—C1 [1.435 (2) Å] bond length, indicating electron delocalization.

The molecules are linked into a chain along the *b* axis by intermolecular C—H···O hydrogen bonds (Fig. 2).

### Experimental

A mixture of the methyl-3-oxobutanoate (5 mmol), 4-ethoxybenzenamine (5 mmol) and InBr_3_ (0.05 mmol) was stirred at room temperature for 1 h. After completion of the reaction, the reaction mixture was diluted with H_2_O (10 ml) and extracted with EtOAc (210 ml). The combined organic layers were dried, concentrated, purified by column chromatography on SiO_2_ with ethyl acetate-cyclohexane (2:8). Single crystals suitable for X-ray diffraction study were obtained from EtOAc-cyclohexane (1:10 *v*/*v*) by slow evaporation at room temperature.

### Refinement

H atoms were placed in geometrically idealized positions and constrained to ride on their parent atoms, with N—H = 0.86 Å, C—H = 0.93–0.97 Å, and *U*_iso_(H) = 1.5*U*_eq_(methyl C) or 1.2*U*_eq_(C,*N*). Each methyl group was allowed to rotate freely about its C—C bond.

### Crystal data

**Table:**

C_13_H_17_NO_3_	*F*_000_ = 504
*M**_r_* = 235.28	*D*_x_ = 1.206 Mg m^−^^3^
Monoclinic, *P*2_1_/*n*	Mo *K*α radiation λ = 0.71073 Å
Hall symbol: -P 2yn	Cell parameters from 2289 reflections
*a* = 12.421 (2) Å	θ = 2.5–24.9º
*b* = 6.3372 (13) Å	µ = 0.09 mm^−^^1^
*c* = 16.569 (3) Å	*T* = 294 (2) K
β = 96.519 (3)º	Block, yellow
*V* = 1295.7 (4) Å^3^	0.30 × 0.26 × 0.20 mm
*Z* = 4	

### Data collection

**Table:**

Bruker SMART CCD area-detector diffractometer	2628 independent reflections
Radiation source: fine-focus sealed tube	1629 reflections with *I* > 2σ(*I*)
Monochromator: graphite	*R*_int_ = 0.031
*T* = 294(2) K	θ_max_ = 26.3º
φ and ω scans	θ_min_ = 2.0º
Absorption correction: multi-scan(SADABS; Sheldrick, 1996)	*h* = −11→15
*T*_min_ = 0.942, *T*_max_ = 0.990	*k* = −7→6
6917 measured reflections	*l* = −20→20

### Refinement

**Table:**

Refinement on *F*^2^	Hydrogen site location: inferred from neighbouring sites
Least-squares matrix: full	H-atom parameters constrained
*R*[*F*^2^ > 2σ(*F*^2^)] = 0.042	*w* = 1/[σ^2^(*F*_o_^2^) + (0.0697*P*)^2^ + 0.1223*P*] where *P* = (*F*_o_^2^ + 2*F*_c_^2^)/3
*wR*(*F*^2^) = 0.136	(Δ/σ)_max_ = 0.001
*S* = 1.00	Δρ_max_ = 0.13 e Å^−^^3^
2628 reflections	Δρ_min_ = −0.11 e Å^−^^3^
158 parameters	Extinction correction: SHELXL97 (Sheldrick, 2008), Fc^*^=kFc[1+0.001xFc^2^λ^3^/sin(2θ)]^-1/4^
Primary atom site location: structure-invariant direct methods	Extinction coefficient: 0.106 (7)
Secondary atom site location: difference Fourier map	

### Special details

**Table:**

Geometry. All e.s.d.'s (except the e.s.d. in the dihedral angle between two l.s. planes) are estimated using the full covariance matrix. The cell e.s.d.'s are taken into account individually in the estimation of e.s.d.'s in distances, angles and torsion angles; correlations between e.s.d.'s in cell parameters are only used when they are defined by crystal symmetry. An approximate (isotropic) treatment of cell e.s.d.'s is used for estimating e.s.d.'s involving l.s. planes.
Refinement. Refinement of *F*^2^ against ALL reflections. The weighted *R*-factor *wR* and goodness of fit *S* are based on *F*^2^, conventional *R*-factors *R* are based on *F*, with *F* set to zero for negative *F*^2^. The threshold expression of *F*^2^ > σ(*F*^2^) is used only for calculating *R*-factors(gt) *etc*. and is not relevant to the choice of reflections for refinement. *R*-factors based on *F*^2^ are statistically about twice as large as those based on *F*, and *R*- factors based on ALL data will be even larger.

### Fractional atomic coordinates and isotropic or equivalent isotropic displacement parameters (Å^2^)

**Table:**

	*x*	*y*	*z*	*U*_iso_*/*U*_eq_	
O1	0.11751 (9)	1.2475 (2)	1.03170 (8)	0.0714 (4)	
O2	0.55147 (10)	0.4131 (2)	0.90568 (7)	0.0697 (4)	
O3	0.60606 (12)	0.2866 (2)	0.79053 (8)	0.0872 (5)	
N1	0.39962 (11)	0.7279 (2)	0.88382 (9)	0.0654 (4)	
H1	0.4403	0.6528	0.9181	0.078*	
C1	0.32646 (13)	0.8715 (3)	0.91658 (9)	0.0578 (4)	
C2	0.21953 (14)	0.8158 (3)	0.92064 (10)	0.0660 (5)	
H2	0.1934	0.6891	0.8979	0.079*	
C3	0.15124 (13)	0.9455 (3)	0.95793 (11)	0.0642 (5)	
H3	0.0794	0.9067	0.9600	0.077*	
C4	0.18978 (13)	1.1339 (3)	0.99243 (10)	0.0562 (4)	
C5	0.29584 (13)	1.1948 (3)	0.98654 (11)	0.0621 (5)	
H5	0.3216	1.3232	1.0079	0.074*	
C6	0.36297 (13)	1.0624 (3)	0.94847 (10)	0.0622 (5)	
H6	0.4341	1.1032	0.9444	0.075*	
C7	0.15753 (16)	1.4153 (3)	1.08352 (12)	0.0747 (6)	
H7A	0.1915	1.5212	1.0526	0.090*	
H7B	0.2113	1.3624	1.1257	0.090*	
C8	0.06476 (18)	1.5100 (4)	1.12071 (12)	0.0860 (6)	
H8A	0.0129	1.5654	1.0787	0.129*	
H8B	0.0908	1.6218	1.1569	0.129*	
H8C	0.0309	1.4036	1.1505	0.129*	
C9	0.41069 (14)	0.6995 (3)	0.80498 (10)	0.0606 (5)	
C10	0.34385 (18)	0.8388 (4)	0.74539 (12)	0.0856 (6)	
H10A	0.2687	0.8250	0.7530	0.128*	
H10B	0.3542	0.7976	0.6911	0.128*	
H10C	0.3660	0.9830	0.7541	0.128*	
C11	0.48003 (16)	0.5549 (3)	0.77912 (11)	0.0682 (5)	
H11	0.4847	0.5450	0.7236	0.082*	
C12	0.54568 (14)	0.4182 (3)	0.83178 (11)	0.0603 (5)	
C13	0.66598 (19)	0.1247 (4)	0.83608 (13)	0.0913 (7)	
H13A	0.7034	0.1842	0.8847	0.137*	
H13B	0.7176	0.0641	0.8038	0.137*	
H13C	0.6171	0.0170	0.8503	0.137*	

### Atomic displacement parameters (Å^2^)

**Table:**

	*U*^11^	*U*^22^	*U*^33^	*U*^12^	*U*^13^	*U*^23^
O1	0.0558 (7)	0.0803 (9)	0.0782 (8)	0.0017 (6)	0.0075 (6)	−0.0156 (7)
O2	0.0708 (8)	0.0769 (9)	0.0602 (8)	0.0030 (6)	0.0030 (6)	−0.0031 (6)
O3	0.1112 (11)	0.0823 (10)	0.0680 (8)	0.0315 (8)	0.0100 (7)	−0.0054 (7)
N1	0.0600 (9)	0.0757 (10)	0.0604 (9)	0.0114 (7)	0.0069 (7)	0.0021 (7)
C1	0.0526 (10)	0.0670 (11)	0.0537 (9)	−0.0002 (8)	0.0050 (7)	0.0037 (8)
C2	0.0572 (11)	0.0703 (12)	0.0701 (11)	−0.0124 (9)	0.0062 (8)	−0.0107 (9)
C3	0.0465 (9)	0.0752 (13)	0.0709 (11)	−0.0097 (8)	0.0067 (8)	−0.0097 (9)
C4	0.0497 (9)	0.0632 (10)	0.0546 (9)	0.0017 (8)	0.0015 (7)	0.0024 (8)
C5	0.0540 (10)	0.0603 (11)	0.0710 (11)	−0.0080 (8)	0.0030 (8)	−0.0018 (9)
C6	0.0481 (10)	0.0686 (12)	0.0698 (11)	−0.0075 (8)	0.0063 (8)	0.0057 (9)
C7	0.0787 (13)	0.0724 (13)	0.0724 (12)	0.0010 (10)	0.0071 (9)	−0.0102 (10)
C8	0.0949 (15)	0.0886 (15)	0.0745 (12)	0.0188 (12)	0.0097 (11)	−0.0090 (11)
C9	0.0589 (10)	0.0621 (11)	0.0613 (10)	−0.0050 (8)	0.0087 (8)	0.0037 (8)
C10	0.0966 (15)	0.0921 (15)	0.0697 (12)	0.0227 (12)	0.0170 (11)	0.0152 (11)
C11	0.0787 (13)	0.0715 (12)	0.0552 (10)	0.0040 (10)	0.0118 (9)	0.0015 (9)
C12	0.0607 (11)	0.0566 (10)	0.0642 (11)	−0.0066 (8)	0.0088 (8)	−0.0060 (9)
C13	0.1021 (16)	0.0811 (15)	0.0877 (15)	0.0275 (12)	−0.0022 (12)	−0.0071 (12)

### Geometric parameters (Å, °)

**Table:**

O1—C4	1.371 (2)	C6—H6	0.93
O1—C7	1.420 (2)	C7—C8	1.493 (3)
O2—C12	1.2187 (19)	C7—H7A	0.97
O3—C12	1.357 (2)	C7—H7B	0.97
O3—C13	1.431 (2)	C8—H8A	0.96
N1—C9	1.341 (2)	C8—H8B	0.96
N1—C1	1.435 (2)	C8—H8C	0.96
N1—H1	0.86	C9—C11	1.360 (3)
C1—C6	1.376 (2)	C9—C10	1.503 (3)
C1—C2	1.383 (2)	C10—H10A	0.96
C2—C3	1.377 (3)	C10—H10B	0.96
C2—H2	0.93	C10—H10C	0.96
C3—C4	1.385 (2)	C11—C12	1.420 (3)
C3—H3	0.93	C11—H11	0.93
C4—C5	1.387 (2)	C13—H13A	0.96
C5—C6	1.384 (2)	C13—H13B	0.96
C5—H5	0.93	C13—H13C	0.96
			
C4—O1—C7	118.48 (14)	H7A—C7—H7B	108.4
C12—O3—C13	117.32 (15)	C7—C8—H8A	109.5
C9—N1—C1	126.37 (15)	C7—C8—H8B	109.5
C9—N1—H1	116.8	H8A—C8—H8B	109.5
C1—N1—H1	116.8	C7—C8—H8C	109.5
C6—C1—C2	118.81 (16)	H8A—C8—H8C	109.5
C6—C1—N1	120.47 (15)	H8B—C8—H8C	109.5
C2—C1—N1	120.63 (16)	N1—C9—C11	122.43 (16)
C3—C2—C1	120.90 (17)	N1—C9—C10	116.80 (16)
C3—C2—H2	119.6	C11—C9—C10	120.76 (16)
C1—C2—H2	119.6	C9—C10—H10A	109.5
C2—C3—C4	119.86 (16)	C9—C10—H10B	109.5
C2—C3—H3	120.1	H10A—C10—H10B	109.5
C4—C3—H3	120.1	C9—C10—H10C	109.5
O1—C4—C3	115.74 (15)	H10A—C10—H10C	109.5
O1—C4—C5	124.43 (16)	H10B—C10—H10C	109.5
C3—C4—C5	119.83 (16)	C9—C11—C12	123.90 (16)
C6—C5—C4	119.30 (17)	C9—C11—H11	118.0
C6—C5—H5	120.4	C12—C11—H11	118.0
C4—C5—H5	120.4	O2—C12—O3	121.13 (16)
C1—C6—C5	121.23 (16)	O2—C12—C11	126.71 (16)
C1—C6—H6	119.4	O3—C12—C11	112.16 (16)
C5—C6—H6	119.4	O3—C13—H13A	109.5
O1—C7—C8	108.47 (16)	O3—C13—H13B	109.5
O1—C7—H7A	110.0	H13A—C13—H13B	109.5
C8—C7—H7A	110.0	O3—C13—H13C	109.5
O1—C7—H7B	110.0	H13A—C13—H13C	109.5
C8—C7—H7B	110.0	H13B—C13—H13C	109.5
			
C9—N1—C1—C6	−100.4 (2)	N1—C1—C6—C5	−174.42 (15)
C9—N1—C1—C2	83.0 (2)	C4—C5—C6—C1	−0.1 (3)
C6—C1—C2—C3	−1.9 (3)	C4—O1—C7—C8	−178.42 (16)
N1—C1—C2—C3	174.69 (16)	C1—N1—C9—C11	−177.96 (17)
C1—C2—C3—C4	−0.4 (3)	C1—N1—C9—C10	3.2 (3)
C7—O1—C4—C3	165.99 (15)	N1—C9—C11—C12	1.1 (3)
C7—O1—C4—C5	−13.3 (2)	C10—C9—C11—C12	179.97 (18)
C2—C3—C4—O1	−176.87 (16)	C13—O3—C12—O2	7.7 (3)
C2—C3—C4—C5	2.5 (3)	C13—O3—C12—C11	−172.85 (18)
O1—C4—C5—C6	177.09 (16)	C9—C11—C12—O2	−1.7 (3)
C3—C4—C5—C6	−2.2 (2)	C9—C11—C12—O3	178.90 (18)
C2—C1—C6—C5	2.2 (3)		

### Hydrogen-bond geometry (Å, °)

**Table:**

*D*—H···*A*	*D*—H	H···*A*	*D*···*A*	*D*—H···*A*
N1—H1···O2	0.86	2.08	2.741 (2)	133
C6—H6···O2^i^	0.93	2.57	3.362 (3)	143

Symmetry codes: (i) *x*, *y*+1, *z*.
